# Long-term outcome of children with acute promyelocytic leukemia: a randomized study of oral versus intravenous arsenic by SCCLG-APL group

**DOI:** 10.1038/s41408-023-00949-w

**Published:** 2023-12-05

**Authors:** Dan-Ping Huang, Liang-Chun Yang, Yi-Qiao Chen, Wu-Qing Wan, Dun-Hua Zhou, Hui-Rong Mai, Wan-Li Li, Li-Hua Yang, He-Kui Lan, Hui-Qin Chen, Bi-Yun Guo, Zi-Jun Zhen, Ri-Yang Liu, Guo-Hua Chen, Xiao-Qin Feng, Cong Liang, Li-Na Wang, Yu Li, Jie-Si Luo, Zhong Fan, Xue-Qun Luo, Bin Li, Yan-Lai Tang, Xiao-Li Zhang, Li-Bin Huang

**Affiliations:** 1 Department of Pediatrics, The First Affiliated Hospital, Sun Yat-sen University, Guangzhou, Guangdong China; 2 Department of Pediatrics, Xiangya Hospital, Central South University, Changsha, Hunan China; 3 Department of Pediatric Hematology, Fujian Medical University Union Hospital, Fuzhou, Fujian China; 4 Department of Pediatrics, Second Xiangya Hospital, Central South University, Changsha, Hunan China; 5 Department of Pediatrics, Sun Yat-sen Memorial Hospital, Sun Yat-sen University, Guangzhou, Guangdong China; 6 Department of Hematology and Oncology, Shenzhen Children’s Hospital, Shenzhen, Guangdong China; 7 Department of Hematology, Hunan Children’s Hospital, Changsha, Hunan China; 8 Department of Pediatrics, Zhujiang Hospital, Southern Medical University, Guangzhou, Guangdong China; 9 Department of Pediatrics, Third Affiliated Hospital, Sun Yat-sen University, Guangzhou, Guangdong China; 10 Department of Pediatrics, First Affiliated Hospital of Xiamen University, Xiamen, Fujian China; 11 Department of Pediatrics, Sun Yat-sen University Cancer Center, Guanzhou, Guangdong China; 12 Department of Pediatrics, Huizhou Central People’s Hospital, Huizhou, Guangdong China; 13 Department of Pediatrics, First People’s Hospital of Huizhou, Huizhou, Guangdong China; 14 Department of Pediatrics, Nanfang Hospital, Southern Medical University, Guangzhou, Guangdong China; 15 Biostatistics Team, Clinical Trials Unit, The First Affiliated Hospital, Sun Yat-sen University, Guangzhou, Guandong China

**Keywords:** Acute myeloid leukaemia, Drug development

## Abstract

Realgar-Indigo naturalis formula (RIF), an oral traditional Chinese medicine mainly containing Realgar (As_4_S_4_), is highly effective in treating adult acute promyelocytic leukemia (APL). However, the treatment efficacy and safety of RIF have not been verified in pediatric patients. SCCLG-APL group conducted a multicenter randomized non-inferiority trial to determine whether intravenous arsenic trioxide (ATO) can be substituted by oral RIF in treating pediatric APL. Of 176 eligible patients enrolled, 91 and 85 were randomized to ATO and RIF groups, respectively. Patients were treated with the risk-adapted protocol. Induction, consolidation, and 96-week maintenance treatment contained all-trans-retinoic acid and low-intensity chemotherapy, and either ATO or RIF. The primary endpoint was 5-year event-free survival (EFS). The secondary endpoints were adverse events and hospital days. After a median 6-year follow-up, the 5-year EFS was 97.6% in both groups. However, the RIF group had significantly shorter hospital stays and lower incidence of infection and tended to have less cardiac toxicity. All 4 relapses occurred within 1.5 years after completion of maintenance therapy. No long-term arsenic retentions were observed in either group. Substituting oral RIF for ATO maintains treatment efficacy while reducing hospitalization and adverse events in treating pediatric APL patients, which may be a future treatment strategy for APL.

## Background

Arsenic-based therapy substantially improves the prognosis of acute promyelocytic leukemia (APL). Besides arsenic trioxide (ATO), another arsenic compound, As_4_S_4_, is also highly effective in the treatment of adult APL. The Realgar-Indigo naturalis formula (RIF), an oral traditional Chinese medicine, contains realgar (As_4_S_4_) as well as Indigo naturalis, Radix salviae miltiorrhizae, and Radix pseudostellariae which yield synergy anti-APL effects [1]. Two multicenter randomized trials have been published till now conducted by one study group (Chinese APL Cooperative Group), confirming that the efficacy of RIF in the treatment of adult APL is not inferior to that of ATO [2, 3]. However, adverse events of ATO and RIF in APL treatment may be different. In adult patients, the RIF group has a higher peak white blood cell count (WBC) compared to the ATO group during induction treatment [4], suggesting a higher potential risk of developing differentiation syndrome (DS) with RIF treatment. On the other hand, RIF had a beneficial effect in accelerating the recovery of thrombocytopenia and hypofibrinogenemia in adults with sub-clinical disseminated intravascular coagulation compared with ATO [5].

The role of RIF in the treatment of children with APL remains less clear. To our knowledge, only one multicenter randomized trial has been published, which reported an interim analysis from our South China Children Leukemia Group (SCCLG) [6], showing that both children with APL in ATO and RIF groups had event-free survival (EFS) of 100%. However, long-term follow-up is needed to better evaluate the efficacy and safety of RIF. It is known that there are important distinctions between pediatric and adult patients with APL [7]. For example, studies suggested that the incidence of leukocytosis (WBC > 10 × 10^9^/L) may be much higher in children with non-high risk (NHR, initial WBC < 10 × 10^9^/L) APL (84%–100%) [8, 9] than in adult counterpart (35%–47%) [10–12] if receiving chemotherapy-free induction treatment with ATO and all-trans retinoic acid (ATRA). Therefore, the SCCLG conducted a randomized study to compare the efficacy and safety between RIF- and ATO-based therapies for the treatment of pediatric APL. We here reported the final results of the trial, which showed that substituting oral RIF for intravenous ATO does not compromise treatment efficacy, but reduces hospital days and adverse events in treating pediatric APL, which have not been observed and reported in adult studies.

## Methods

### Study design and patients

The SCCLG-APL study was a multicenter, prospective, randomized, non-inferiority trial comparing the efficacy and safety of oral RIF and intravenous ATO in the treatment of pediatric APL. The study received institutional review board approval. Patients’ families and/or patients provided informed consent in accordance with the Declaration of Helsinki. The trial was registered at www.clinicaltrials.gov as NCT02200978.

Patients were recruited from 14 participating hospitals from September 2011 to July 2020. Eligible patients were 16 years old or younger with newly diagnosed APL with confirmation of PML-RARa by reverse-transcription PCR (RT-PCR) assay and (or) fluorescence in situ hybridization. Those were excluded if they had one of the following events occurring before randomization: death from any cause, or coma, convulsion, paralysis due to intracranial hemorrhage, cerebral thrombosis or central nervous system leukemia; or who had prolonged QT syndrome because of the risk of QT interval prolongation during arsenic therapy; or who did not accept randomization.

In this open-label trial, patients were randomly assigned 1:1 by computer-generated codes to the ATO or RIF group. The last follow-up time was May 31, 2023. The primary endpoint was 5-year EFS. Secondary end-points were the number of cumulated hospital days and adverse events including incidences of leukocytosis, DS, and cardiac events during induction and consolidation treatment.

### Treatment protocol

Patients were treated with a risk-adapted protocol (Suppl. Table S1) which had been published [6]. Briefly, patients received oral ATRA once they were suspected as APL based on morphological features. Mitoxantrone was administrated on day 3 (10 mg/m^2^) for low-risk (LR, initial WBC count ≤10 × 10^9^/L and platelet count ≥40 × 10^9^/L) and intermediated-risk (IR, initial WBC count ≤10 × 10^9^/L and platelet count <40 × 10^9^/L) or on days 2–4 (7 mg/m^2^/day) for high-risk (HR, initial WBC count >10 × 10^9^/L) patients, respectively. When the diagnosis was genetically confirmed (5 days were needed for all patients including those admitted on Fridays and holidays), patients were randomly assigned to ATO or RIF groups. In the ATO group, ATO was administrated at 0.16 mg/kg/day (≯10 mg/day) intravenously over 12 h until hematologic complete remission (HCR) was achieved and then followed by three courses of consolidation therapy containing ATRA, ATO, and low-intensity chemotherapy (CHT) with mitoxantrone and addition of cytarabine for HR patients. Maintenance therapy included ATRA, ATO, methotrexate, and 6-mercaptopurine. In the RIF group, ATO was replaced by RIF (270 mg with Realgar 30 mg per pill). RIF was given at 135 mg/kg/day (≯30 pills (8100 mg)/day) orally three times daily. Intrathecal injection of cytarabine and dexamethasone was administrated on day 1 of every course of consolidation therapy.

Measurable residual disease (MRD) monitoring of bone marrow was performed by Quantitative reverse-transcription PCR (qRT-PCR) for PML/PARα (the fusion of promyelocytic leukemia (PML) gene and retinoic acid receptor alpha (RARA) genes) at the end of induction, before the beginning of maintenance, and every 24 weeks from the beginning of maintenance to 48 weeks after the end of maintenance (Suppl. Table S1). For evaluating adverse events during induction and consolidation therapy, routine blood tests and coagulation function tests were performed from once daily or more to once every other day according to doctor’s judgment based on clinical conditions; liver and kidney function blood tests, and electrocardiograms were carried out before the beginning and after the end of each course, while echocardiography and serum creatine Kinase MB isoenzyme (CK-MB) or troponin I (Tn-I) assay were performed before the beginning of each course.

### Supportive therapy

When patients’ WBC count was over 10 × 10^9^/L at diagnosis or during induction treatment, hydroxyurea at 100 mg/kg/day was given until WBC < 10 × 10^9^/L. Dexamethasone at 0.3 mg/kg/day was given if DS or ATRA-associated pseudotumor cerebri was suspected. Vigorous blood product transfusion was given for the aims of maintaining platelet counts (PLT) over 30 × 10^9^/L and fibrinogen levels greater than 1.5 g/L, respectively.

### Urine arsenic excretion rate

The ratio of arsenic to creatinine (uAs_Cr_, μmol/mmol uCr) in the same spot urinary sample was used to assess the urine arsenic excretion rate. The method used for the testing has been reported in our recent paper [13]. We used spot urine samples for assessing urine arsenic excretion because collecting 24 h urine samples is not practical in children. Urine arsenic excretion rates were tested at the following time points: before the use of arsenic treatment, 1–3 days before the beginning of maintenance therapy, and 1–7 days after the end of maintenance.

### Statistical analysis

The primary endpoint was the 5-year EFS, and the analysis was per protocol. Assuming a 95% rate of 5-year EFS for children with APL treated on arsenic-ATRA-CHT protocol based on the data from a trial of adult patients [2], a non-inferiority margin of -10%, 5% type I error (one-sided) and 90% power, 82 evaluable patients per group (164 in total) were required to draw a non-inferiority conclusion. Non-inferiority was concluded if the lower limit of the 95% CI for the rate difference of EFS was greater than –10% non-inferiority margin. The Kaplan–Meier method was used to estimate overall survival (OS; time from date of randomization to death) and EFS (time from date of randomization to last follow-up or first event including failure to achieve HCR, hematologic/molecular relapse, secondary malignancy or death of any cause). Patients lost to follow-up were censored at their date of last known contact. The significance of predictor variables was tested by the log-rank statistic for survival rates. Mean ± SD was used to describe normally distributed variables, while skewed variables were expressed as median and range or interquartile range (IQR). The two independent samples *t*-test and Mann–Whitney *U-*test were used for normally distributed data and skewed data to compare between groups separately. The comparison between categorical variables was evaluated by the Chi-square (*χ*^2^) test. Data were analyzed with the use of the statistical packages R (The R Foundation, version 4.2.0) and Empower (R), SPSS Statistics version 27.00 (IBM SPSS Statistics, Armonk, NY), and GraphPad Prism version 8.0 (GraphPad Software).

## Results

### Enrollment and patient characteristics

**Fig. 1:**
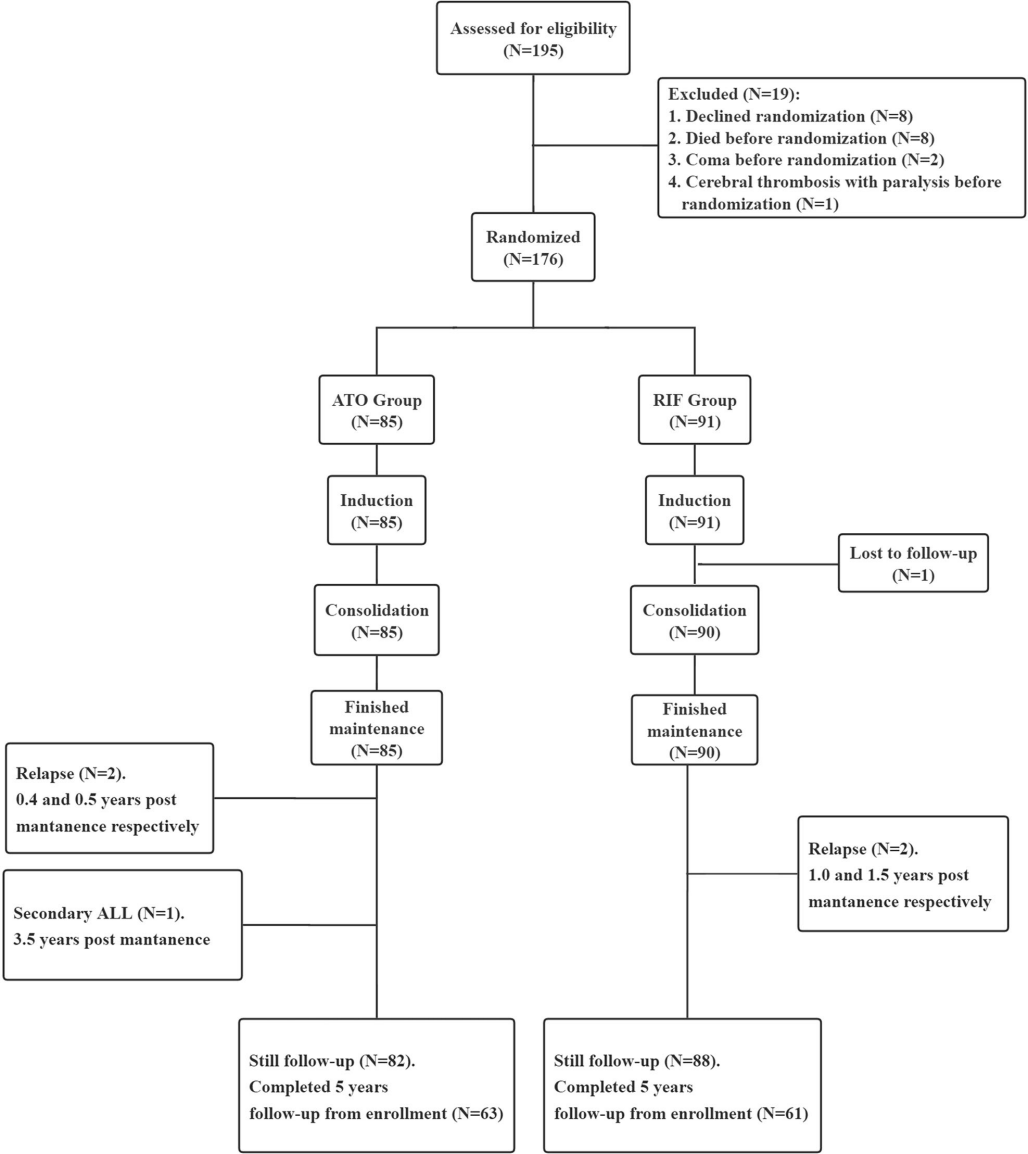
CONSORT diagram of enrolled patients. ATO arsenic trioxide, RIF Realgar-Indigo naturalis formula, ALL acute lymphoblastic leukemia.

A total of 204 patients were morphologically diagnosed with APL, of whom 9 were PML-RARa negative. Among the remaining 195 patients, 19 were excluded because 11 died or met the exclusion criteria before randomization and 8 declined randomization (Fig. 1 and Suppl. Table S2). Therefore, 176 were enrolled and randomly assigned to the ATO or RIF group (Fig. 1). There were no significant differences in the baseline characteristics between the two groups (Table 1).

**Table 1:** The basic characteristics of eligible patients in ATO and RIF groups.

	Total *N* = 176	ATO groups *N* = 85	RIF groups *N* = 91	*p*
Median age, years (range)	8.2 (1.0,15.9)	7.9 (1.0,14.6)	8.8 (1.5,16.6)	0.613
Male, *n* (%)	105 (59.7)	57 (67.1)	48 (52.7)	0.053
Median WBC, ×10^9^/L (range)	4.5 (0.3,228.0)	4.4 (0.3,228.0)	4.8 (0.8,74.7)	0.876
Median PLT, ×10^9^/L (range)	25.5 (4.0,226.0)	26.5 (4.0,135.0)	24.0 (4.0,226.0)	0.723
Median Hb, g/L (range)	74.5 (31.0,141.0)	74.0 (31.0,131.0)	76.0 (37.0,141.0)	0.321
Sanz risk, *n* (%),				
Low-risk	34 (19.3)	15 (17.6)	19 (20.9)	0.804
Intermediate-risk	85 (48.3)	43 (50.6)	42 (46.2)	
High-risk	57 (32.4)	27 (31.8)	30 (32.9)	
CNSL at diagnosis	0	0	0	

*WBC* white blood cell count, *PLT* platelet counts, *Hb* hemoglobin level, *Low-risk APL* WBC ≤ 10 × 10^9^/L and PLT ≥ 40 × 10^9^/L, *intermediate-risk* WBC ≤ 10×10^9^/L and PLT < 40 × 10^9^/L, *high-risk* WBC > 10 × 10^9^/L at diagnosis, *CNSL* central nervous system leukemia defined as having clinical signs of facial nerve palsy, hypothalamic syndrome, etc., and/or MRI evidence of intracranial, intradural mass.

### Efficacy

All of the 176 eligible patients achieved HCR after induction therapy. The median (IQR) time to HCR was 25.0 (21.0–30.0) days in the ATO group and 27.0 (22.0–35.0) days in the RIF group (*p* = 0.082). One IR patient in the RIF group achieved molecular complete remission (MCR, qRT-PCR negative for PML-RARa) after induction treatment and later was lost to follow-up. All the remaining 175 patients, 85 in ATO and 90 in RIF groups achieved MCR at the end of consolidation.

**Fig. 2:**
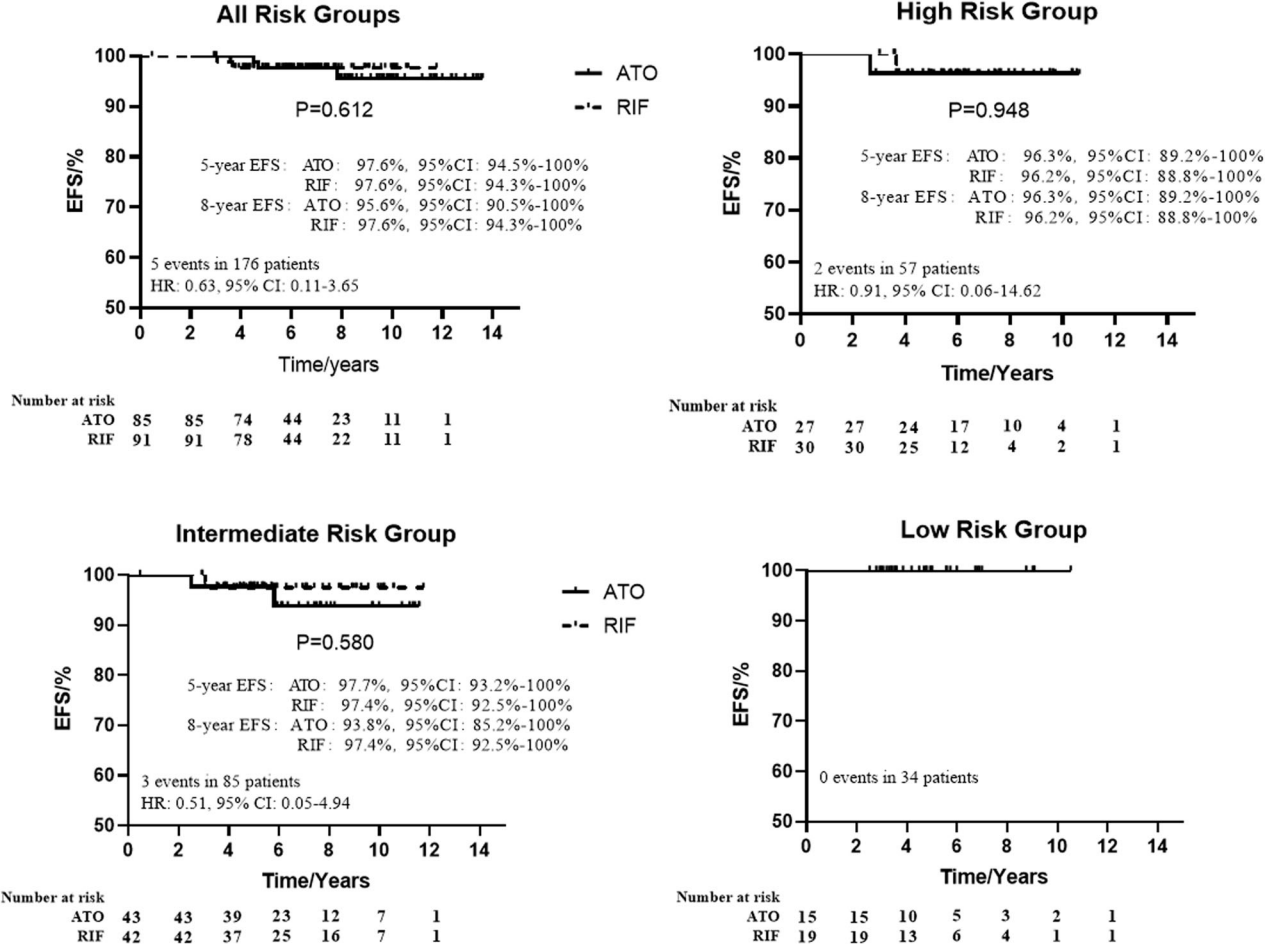
Event-free survivals of ATO and RIF groups. ATO arsenic trioxide, RIF Realgar-Indigo naturalis formula, EFS event-free survivals, HR Hazard Ratio, CI confidence interval.

In all of the 176 patients with a median follow-up of 6 years (95% CI, 5.7–6.3), the 8-year OS was 100%, and the 5- and 8-year EFS were 97.7% (95% CI, 95.3%–100.0%) and 96.6% (95% CI, 93.7%–99.5%), respectively. The 5-year EFS in ATO and RIF groups were both 97.6% (*p* = 0.612) (Fig. 2). The difference in 5-year EFS between the two groups was 0% (95% CI, –0.038% to 0.038%). The lower limit of the 95% CI for the percentage difference in 5-year EFS was greater than the –10% non-inferiority margin, confirming non-inferiority (*p* = 0.000007). There was no significant difference in EFS between LR, IR, and HR patients according to Sanz risk stratification (*p* = 0.548).

### Safety

Treatment-related adverse events are listed in Tables 2 and 3. Toxic events were graded according to the National Cancer Institute’s Common Toxicity Criteria (version 5.0).

**Table 2:** Adverse events during induction treatment, *n* (%).

	Total *N* = 176	ATO *N* = 85	RIF *N* = 91	*p*
Differentiation syndrome				
Moderate	11 (6.3)	7 (8.2)	4 (4.4)	0.293
Severe	1 (0.6)	1 (1.2)	0 (0)	0.483
Infection/FUO	134 (76.1)	65 (76.5)	69 (75.8)	0.920
PCT	28 (15.9)	13 (15.3)	15 (16.5)	0.829
Coagulopathy events				
Hemorrhage	8 (4.5)	3 (3.5)	5 (5.5)	0.721
Thrombosis	3 (1.7)	3 (3.5)	0 (0)	0.111
Liver				
Grade 1–2	19 (10.8)	11 (12.9)	8 (8.8)	0.375
Grade 3–4	0 (0)	0 (0)	0 (0)	-
Cardiac				
Grade 1–2	1 (0.6)	1 (1.2)	0 (0)	0.483
Grade 3–4	1 (0.6)	1 (1.2)	0 (0)	0.483
Gastrointestinal reaction				
Grade 1–2	49 (27.8)	25 (29.4)	24 (26.4)	0.653
Grade 3–4	0 (0)	0 (0)	0 (0)	-
Rash	11(6.3)	5 (5.9)	6 (6.6)	0.845
Hyperlipemia	1(0.6)	0 (0)	1 (1.1)	1.000

*FUO* fever of unknown origin, *PCT* pseudotumor cerebri.

**Table 3:** Adverse events during consolidation treatment, *n* (%).

	Total *N* = 175	ATO *N* = 85	RIF *N* = 90	*p*
Infection/FUO	60 (34.3)	36 (42.4)	24 (26.7)	0.029
PCT	48 (27.4)	29 (34.1)	19 (21.1)	0.063
Coagulopathy events				
Hemorrhage	7 (4.0)	5 (5.9)	2 (2.2)	0.267
Thrombosis	2 (1.1)	1 (1.2)	1 (1.1)	1.000
Liver				
Grade 1–2	5 (2.9)	2 (2.4)	3 (3.3)	1.000
Grade 3–4	0 (0)	0 (0)	0 (0)	-
Cardiac^a^				
Grade 1–2	3 (1.7)	3 (3.5)	0 (0)	0.112
Grade 3–4	1 (0.6)	1 (1.2)	0 (0)	0.486
Gastrointestinal dysfunction				
Grade 1–2	27 (15.4)	11 (12.9)	16 (17.8)	0.376
Grade 3–4	0 (0)	0 (0)	0 (0)	-
Rash	2 (1.1)	1 (1.2)	1 (3.3)	1.000

*FUO* fever of unknown origin, *PCT* pseudotumor cerebri.

^a^The difference in the total incidence of cardiac events (grade 1–4) between the ATO and RIF groups was close to being significant (*p* = 0.054).

During induction treatment, 50 (42.0%) of the NHR patients, 23 (39.7%) in the ATO group, and 27 (44.3%) in the RIF group (*p* = 0.611) developed leukocytosis greater than 10 × 10^9^/L; and 28 (49.1%) of the HR patients, 14 (51.9%) in ATO group and 14 (46.7%) in RIF group (*p* = 0.696), experienced a WBC count increase by more than 30% as compared with that at diagnosis, respectively. Eight (9.4%) and four (4.4%) patients developed DS (including moderate and severe forms) in ATO and RIF groups, respectively (Table 2 and Suppl. Table S3), and the incidence was not statistically different between the two groups. Other adverse events were not severe (Table 2). All the adverse events were successfully managed as per protocol recommendation.

During consolidation, the incidence of infection or fever of unknown origin (FUO) was significantly lower in the RIF group than in the ATO group. Moreover, the total incidence of cardiac toxicity in the RIF group was also lower than in the ATO group and the difference was close to being statistically significant (*p* = 0.054) (Table 3 and Suppl. Table S4).

### Hospital days

Except for the patient lost to follow-up after induction, the mean cumulated days required for inpatient management during induction and consolidation therapy were significantly less in RIF than in the ATO group whatever the children with LR, IR, or HR APL (Table 4).

**Table 4:** Cumulated hospital days during induction and consolidation therapy, median (IQR).

	Hospital days		
	ATO	RIF	*p*
High-risk	68.0 (45.0,77.5)	44.5 (40.0,63.0)	0.048
Intermediate-risk	64.0 (46.5,73.5)	39.0 (28.0,49.0)	0.000
Low-risk	65.0 (56.0,76.5)	34.0 (24.0,47.5)	0.000

*Low-risk APL* WBC ≤ 10 × 10^9^/L and PLT ≥ 40 × 10^9^/L, *intermediate-risk* WBC ≤ 10 × 10^9^/L and PLT < 40 × 10^9^/L, *high-risk* WBC > 10 × 10^9^/L at diagnosis.

### Arsenic retention on follow-up

The urine arsenic excretion rates at different treatment time points are shown in Table 5 and were significantly elevated after induction and consolidation therapy (before maintenance). At the end of maintenance therapy, the median excretion rate returned to base level in both the two groups. There was no statistically significant difference in the excretion rates between the two groups.

**Table 5:** Excretion rates of urine arsenic (uAs_Cr_, μmol/mmol), median (IQR).

	Total	ATO group	RIF group	*p*
Before arsenic	*N* = 139 0.0140 (0.0082, 0.0366)	*N* = 69 0.0160 (0.0104, 0.0385)	*N* = 70 0.0128 (0.0066, 0.0343)	0.098
Before maintenance	*N* = 136 0.1441^a^ (0.0941, 0.3869)	*N* = 66 0.1423 (0.1062, 0.3918)	*N* = 70 0.1481 (0.0750, 0.3766)	0.418
End of maintenance	*N* = 72 0.0177^b^ (0.0104, 0.0274)	*N* = 37 0.0180 (0.0114, 0.0286)	*N* = 35 0.0158 (0.0098, 0.0253)	0.297

Compare with the excretion rates of urine arsenic before arsenic treatment: ^a^*p* < 0.001, ^b^*p* < 0.05.

We compared urine arsenic excretion rates between relapsed and non-relapsed patients (Suppl. Tables S5), and found that the median excretion rate before the beginning of maintenance therapy tended to be lower in the former than in the latter (*p* = 0.090).

### Relapse and secondary malignancy

Four patients relapsed, two in ATO and two in RIF groups, between 0.4 and 1.5 years after the completion of maintenance treatment (Suppl. Table S6). There were no FLT3-ITD mutations and positive expressions of CD56, CD34, and CD2 in APL cells found in all of the 4 relapsed both at diagnosis and relapse. One IR patient in the ATO group developed acute lymphoblastic leukemia after 42.6 months of the completion of maintenance treatment, received allogeneic stem cell transplantation, and remained in complete remission for 44 months till the last follow-up visit in May 2023.

## Discussion

To our knowledge, only one study group (Chinese APL Cooperative Group) has reported multicenter randomized studies comparing RIF and ATO in adult APL [2, 3]. This study is the first multicenter and randomized trial comparing oral RIF and intravenous ATO in the treatment of pediatric APL. With a median 6-year follow-up, children with APL in all risks treated on SCCLG-APL protocol had long-term OS and EFS of 100% and 96.6%, respectively, at 8 years, and the EFS were not significantly different between the two groups regardless of risk stratification at diagnosis.

The incidence of infection or FUO was significantly lower in the RIF group. RIF can be taken orally in an outpatient setting when the disease is stable, which decreases the risk of cross-infection in the hospital and iatrogenic infection due to PICC line placement, altogether resulting in shorter hospital stays. Moreover, the total incidence of cardiac adverse events during consolidation tended to be lower in the RIF group than in the ATO group (*p* = 0.054) (Table 3 and Suppl. Table S4), which supports the result obtained by a retrospective study in children [14]. However, the advantages of less infection and cardiac toxicity in APL treatment with RIF than with ATO were not observed in trials conducted in adult patients [2, 3], which again highlights the distinctions between pediatric and adult patients with APL.

Treatment intensity can be further reduced in the arsenic and ATRA era, as reported recently that chemotherapy-free treatment was used for patients with NHR APL, and intrathecal may not be required for those without central nervous system leukemia or intracerebral hemorrhage [3, 11, 12, 15, 16]. There is concern that the use of the two differentiating agents (arsenic and ATRA) without chemotherapy in induction may result in an increasing risk of leukocytosis and differentiation syndrome [17]. Previous studies suggested that the incidence of leukocytosis (>10 × 10^9^/L) in pediatric patients with NHR APL was 84%–100% [9, 10] and much higher than 35%–47% [10–12] occurred in adult counterpart, if treated with the CHT-free induction. One study reported CHT-free induction treatment with ATRA and arsenic in children with NHR APL and administering anthracycline only to those with HR APL [15], the total incidence of DS was 41% and much higher than the 6.8% in our cohort (Suppl. Table S3). Another clinical trial also conducted in children with NHR APL showed that the CHT-free induction resulted in 24.5% (24/98) of children having DS [16]. Of those having DS, 70.8% and 50.0% had respiratory distress and hypoxemia, respectively, 16.7% to 29.2% had pulmonary infiltrates, pleural effusion, and hypotension, 8.3% had acute renal failure, and one died of DS. Therefore, to investigate the safety of the CHT-free induction in children with NHR APL, SCCLG-APL has initiated a new randomized study to compare induction treatments with and without an addition of one dose of anthracycline to ATRA and arsenic.

Arsenic retention is another major concern, especially in children treated with arsenic. To our knowledge, there has been no study comparing arsenic retention in children between treatments with RIF and ATO. A single-arm study investigated arsenic levels in plasma, hair, nail, and urine at different treatment time points in children with APL treated with ATO or RIF, and showed that the arsenic levels of the first three (except urine) returned to normal after 6 months stopping arsenic administration [15]. In this study, we used urinary arsenic excretion rate (urine arsenic is adjusted by the creatinine) to assess arsenic retention because it is better than using urine arsenic alone [18], and is positively correlated with plasma arsenic levels as reported in our previous investigation [13]. The present study showed that at the end of maintenance therapy, the median arsenic excretion rate returned to base level in both RIF and ATO groups. Thus, it is not likely to result in long-term arsenic retention regardless of treatment with ATO or RIF in children.

Relapse is uncommon in APL following contemporary treatment. This study proved that FLT3-ITD mutations are not a risk factor for relapse of pediatric APL, which is in line with the results of recent studies observed in adult patients [19]. Our previous studies showed that ATO/ATRA combination can work synergistically to promote ubiquitination-mediated and autophagic degradation of FLT3-ITD protein, selectively kill FLT3-ITD leukemia cells, and reduce the leukemic burden in mice with FLT3-ITD leukemia [20–22]. These experimental studies may explain, at least in part, how ATO and ATRA-based therapy can abrogate the negative impact of FLT3-ITD mutations. Some studies mainly on adult patients suggested that CD56+, CD34+, or CD2+CD34+APL subgroup had a higher rate of relapse [23–25], however, this was not proved in our pediatric patients treated on SCCLG-APL protocol. We also observed that urine arsenic excretion rate tended to be lower in relapsed than in non-relapsed patients (*p* = 0.090) (Suppl. Tables S5), indicating plasma arsenic level was correspondingly lower in the former [13]. As there were only 4 relapses in our cohort, the sample was too small to draw a conclusion that the relapse of the disease is related to insufficient plasma concentration of arsenic. Nevertheless, it is necessary to investigate the relationship between the relapse and plasma arsenic levels in the future. Lastly, there has been no clear guideline till now for how long should MRD be monitored in APL. This long-term study indicated that it is not necessary for MRD monitoring for more than 1.5 years after completion of maintenance therapy if MCR is achieved at the end of consolidation. Given the very low relapse rate of APL with contemporary treatment, MRD monitoring after MCR is only recommended for HR patients [19]. For NHR patients, using a peripheral blood sample instead of bone marrow for MRD monitoring may be considered if needed.

Although we obtained an excellent long-term survival rate in our cohort, there were 10 of 195 (5.1%) patients died before randomization. Among them, 8 died of intracranial hemorrhage, 1 intracranial hemorrhage and pulmonary hemorrhage, and 1 alimentary tract hemorrhage (Suppl. Table S2). The risk of hemorrhagic death during the induction period remains at about 5% in the clinical trial setting even nowadays [26, 27], and the mortality rate before diagnosis is unknown. Therefore, the survival rate of APL patients is lower in the real world. Although not all patients have completed the 5 years of follow-up from enrollment in this report (Fig. 1), the non-inferiority of RIF in efficacy compared with ATO was demonstrated, and both groups had 5-year EFS of 97.6%, after a median follow-up of 6 years. Another important observation was that oral RIF treatment can significantly reduce hospital days and incidences of adverse events.

In conclusion, the final results of the multicenter randomized trial SCCLG-APL confirmed that oral RIF is as effective as intravenous ATO for the treatment of pediatric APL. The substitution of RIF for ATO has the additional advantages of shortened hospital days, less infection risk, and cardiac toxicity in pediatric patients. Given its advantage over ATO in treating APL patients across all age groups, promoting the wider use of oral RIF outside of China is worthwhile.

### Supplementary information

SUPPLEMENTAL MATERIAL

## Data Availability

The data that support the findings of this study are available on request from the corresponding author upon reasonable request.
