# Supplementary material for: Long-term outcome of children with acute promyelocytic leukemia: a randomized study of oral versus intravenous arsenic by SCCLG-APL group
Source: Blood Cancer J. 2023 Dec 5;13(1):178. doi: 10.1038/s41408-023-00949-w (PMC10698191; doi:10.1038/s41408-023-00949-w)
Supplement: Supplementary file 1 — SUPPLEMENTAL MATERIAL [file 41408_2023_949_MOESM1_ESM.docx]

**Appendix**

| Table S1. SCCLG-APL protocol |
| --- |
| **Induction** |
| ATRA 25mg/m^2^/d, orally 2-3 times daily, d1-HCR^&^ (maximum 42 days) |
| MA 10mg /m^2^/d, intravenously, d3 (low- and intermediated-risk)  7mg /m^2^/d, intravenously, d2-4 (high-risk) |
| ATO* 0.16mg/kg/d (maximum 10mg/day), intravenously over 12 hours, d5-HCR^&^ |
| **Consolidation 1** |
| ATRA 25mg/m^2^/d, orally 2-3 times daily, d1-15 |
| MA 10mg /m^2^/d, intravenously, d1-2 |
| **Consolidation 2^#^** |
| ATO* 0.16mg/kg/d (maximum 10 mg/day), intravenously over 12 hours, d1-15 |
| AC 1g/m^2^/d, intravenously, d1-2 (high-risk only) |
| **Consolidation 3^#^** |
| ATRA 25mg/m^2^/d, orally 2-3 times daily, d1-15 |
| ATO* 0.16mg/kg/d (≯10 mg/day), intravenously over 12 hours, d1-15 |
| MA 10mg /m^2^/d, intravenously, d1 |
| AC 1g/m^2^/d, intravenously, d1-2 (high-risk only) |
| **Maintenances (cycle 1)^** |
| ATRA 25mg/m^2^/d, orally 2-3 times daily, d1-14 |
| ATO* 0.16mg/kg/d (maximum 10 mg/day), intravenously over 12 hours, d1-14 |
| MTX 20mg/m^2^/week, d15-63 |
| 6MP 50mg/m^2^/d, d15-63 |
| **Maintenances (cycle 2)^** |
| ATRA 25mg/m^2^/d, orally 2-3 times daily, d1-14 |
| MTX 20mg/m^2^/week, orally, d15-63 |
| 6MP 50mg/m^2^/d, orally, d15-63 |
| Abbreviations: ATRA, all-trans-retinoic acid; ATO, arsenic trioxide; MA, mitoxantrone;AC, cytarabine; MTX, methotrexate; 6MP, 6-mercaptopurine.  ^&^HCR, haematologic complete remission; *Patients in Realgar-Indigo naturalis formula (RIF) group received RIF at 135 mg/kg/d (maximum 30 pills/d) orally three times daily instead of receiving ATO. **^#^**The interval between consolidations 1, 2, 3 and the beginning of first cycle 1 of maintenance was 28 days. ^Repeated cycles 1 and 2 for a total of 8 cycles.  Confirmation of diagnosis of PML-RARa positive APL can be made on day 5 of ATRA induction treatment for all patients with suspected APL, even for those admitted on Friday and holidays. The genetically diagnosed patients were randomly assigned (1:1) by computer-generated codes to ATO or RIF group.  Intrathecal injection of cytarabine and dexamethasone was administrated on day 1 of every course of consolidation therapy.  Minimal residual disease monitoring of bone marrow was performed by qRT-PCR for PML/PARα at the end of induction, before the beginning of maintenance and every 24 weeks from the beginning of maintenance to 48 weeks after the end of maintenance. |

Table S2. Basic characteristics of excluded patients having events before randomization

| Gender | Age/years | Sanz risk | events | ATRA^*^ | Outcomes^#^ |
| --- | --- | --- | --- | --- | --- |
| Female | 7.8 | HR | Intracranial hemorrhage | Yes | Died at D5 |
| Female | 9.0 | HR | Intracranial hemorrhage | Yes | Died at D3 |
| Female | 14.3 | IR | Alimentary tract hemorrhage | Yes | Died at D5 |
| Male | 6.4 | IR | Intracranial hemorrhage, pneumorrhagia | No | Died at D3 |
| Female | 13.9 | HR | Intracranial hemorrhage | Yes | Died at D4 |
| Male | 13.8 | HR | Intracranial hemorrhage | Yes | Died at D5 |
| Female | 13.8 | HR | Intracranial hemorrhage | Yes | Died at D15^ |
| Female | 3.6 | HR | Intracranial hemorrhage | Yes | Died at D9^&^ |
| Female | 4.0 | HR | Intracranial hemorrhage | Yes | Died at D4 |
| Male | 6.9 | HR | Intracranial hemorrhage | Yes | Died at D4 |
| Female | 14.1 | HR | Cerebral thrombosis and paralysis | Yes | CCR^$^ |

CCR: Continuous completed remission. ^*^ATRA was given within 3 days of hospitalization when APL was suspected. ^#^Days of death after hospitalization. ^Convulsion and coma on day 5 of hospitalization. ^&^Coma on day 3 of hospitalization. ^$^She had left hemiplegia before admission, and was treated with SCCLG-APL protocol (ATO) and now is in CCR for 7.3 years.

Table S3. Symptoms of differentiation syndrome occurred during induction treatment

| Characteristis | All (N=176) | | NHR (N=119) | | | HR (N=57) | |
| --- | --- | --- | --- | --- | --- | --- | --- |
|  | N | % | | N | % | N | % |
| Total | 176 |  | | 119 |  | 57 |  |
| DS |  |  | |  |  |  |  |
| No | 164 | 93.2 | | 114 | 95.8 | 50 | 87.7 |
| Yes | 12 | 6.8 | | 5 | 4.2 | 7 | 12.3 |
| DS: Fever |  |  | |  |  |  |  |
| No | 0 | 0.0 | | 0 | 0.0 | 0 | 0.0 |
| Yes | 12 | 100.0 | | 5 | 100.0 | 7 | 100.0 |
| DS: Respiratory distress |  |  | |  |  |  |  |
| No | 6 | 50.0 | | 3 | 60.0 | 3 | 42.9 |
| Yes | 6 | 50.0 | | 2 | 40.0 | 4 | 57.1 |
| DS: Pulmonary infiltrates |  |  | |  |  |  |  |
| No | 6 | 50.0 | | 3 | 60.0 | 3 | 42.9 |
| Yes | 6 | 50.0 | | 2 | 40.0 | 4 | 57.1 |
| DS: Hypotension |  |  | |  |  |  |  |
| No | 9 | 75.0 | | 4 | 80.0 | 5 | 71.4 |
| Yes | 3 | 25.0 | | 1 | 20.0 | 2 | 28.6 |
| DS: Serous effusion |  |  | |  |  |  |  |
| No | 9 | 75.0 | | 3 | 60.0 | 6 | 85.7 |
| Yes | 3 | 25.0 | | 2 | 40.0 | 1 | 14.3 |
| DS: Congestive heart failure |  |  | |  |  |  |  |
| No | 11 | 91.7 | | 5 | 100.0 | 6 | 85.7 |
| Yes | 1 | 8.3 | | 0 | 0.0 | 1 | 14.3 |

DS: differentiation syndrome. NHR: non-high-risk. HR: high-risk.

Table S4. Cardiac adverse events

LR: low-risk; IR: intermediate-risk; HR: high-risk; CK-MB: creatine kinase-MB; pro-BNP: pro brain

| Cases | Group | Sanz risk | Period | Grade | Clinical manifestation |
| --- | --- | --- | --- | --- | --- |
| 1 | ATO | IR | Induction | 1-2 | Asymptomatic Q-T interval prolongation |
| 2 | ATO | HR | Induction | 3-4 | Chest pain, Traube’s bruit |
| 1 | ATO | IR | Consolidation | 1-2 | Left axis deviation |
| 2 | ATO | IR | Consolidation | 1-2 | Transient chest pain |
| 3 | ATO | LR | Consolidation | 1-2 | Transient elevated CK-MB and pro-BNP |
| 4 | ATO | LR | Consolidation | 3-4 | Elevated CK-MB and delayed discharge |

natriuretic peptide.

Table S5. Urine arsenic excretion rates between relapsed and non-relapsed patients,

(uAsCr, μmol/mmol), median (IRQ)

|  | Total | Relapsed group | Non-relapsed groupgroup | P value |
| --- | --- | --- | --- | --- |
| Before treatment | N=139  0.0140  (0.0083，0.0366) | N=4  0.0202  (0.0092，0.0342) | N=135  0.0140  (0.0083，0.0366) | 0.950 |
| 1- 3 days before maintenance | N=136  0.1441  (0.0941，0.3869) | N=4  0.0941  (0.0688，0.1114) | N=132  0.1481  (0.0948，0.3911) | 0.090 |

Table S6. Characteristics of four relapsed patients

| Case | 1 | 2 | 3 | 4 |
| --- | --- | --- | --- | --- |
| Treatment group | RIF | ATO | RIF | ATO |
| Sex | Male | Female | Male | Male |
| Age of years at diagnosis years | 13.2 | 14.6 | 4.2 | 2.8 |
| Sanz risk | HR | IR | IR | HR |
| Bcr subtype | bcr1 | bcr1 | bcr1 | bcr1 |
| CD2 | - | - | - | - |
| CD34 | - | - | - | - |
| CD56 | - | - | - | - |
| FLT3-ITD mutations | - | - | - | - |
| MRD after induction | + | + | + | + |
| MRD after consolidation | - | - | - | - |
| Years post-maintenance | 1.5 | 0.4 | 1.0 | 0.5 |
| Outcome | *In MCR2  for 4 years | ^#^In MCR2 for  3.1 years, then in CR3 for 2 months | *In MCR2  for 3.5 years | *In MCR2  for 3.8 years |
| IR: intermediate-risk; HR: high-risk. -: negative; +: positive; MRD: minimal Residual Disease assessed by qRT-PCR. MCR: molecular complete remission. *Be treated with SCCLG protocol for relapsed APL. ^#^Be treated with other protocol in a hospital outside the SCCLG and relapsed again 3.1 years later, now is in hematological CR3 and waiting for stem cell transplantation. | | | | |
